# The outcome of young vs. old gastric cancer patients following gastrectomy: a propensity score matching analysis

**DOI:** 10.1186/s12893-021-01401-1

**Published:** 2021-11-19

**Authors:** Yu-Xi Cheng, Wei Tao, Xiao-Yu Liu, Chao Yuan, Bin Zhang, Wei Zhang, Dong Peng

**Affiliations:** Department of Gastrointestinal Surgery, The First Affiliated Hospital of Chongqing Medical University, Chongqing, 400016 China

**Keywords:** Age, Gastric cancer, Propensity score matching, Overall survival, Disease-free survival

## Abstract

**Purpose:**

The purpose of the current study was to compare the postoperative complications, overall survival and disease-free survival in young and old gastric cancer patients after gastrectomy using propensity score matching (PSM).

**Methods:**

Adult patients (aged ≥ 18 years) who underwent gastrectomy for gastric cancer in a single clinical center from January 2013 to December 2017 were enrolled continuously for retrospective analysis. To minimize the selection bias between the young and old groups, the PSM was conducted in this study.

**Results:**

A total of 558 patients were included in this study, with 51 patients in the young group (aged ≤ 45 years) and 507 patients in the old group (aged > 45 years). After 1:1 matching according to PSM, 51 patients in the young group were matched to 51 patients in the old group. After PSM, there was no difference in the baseline information. In terms of short-term outcomes, no difference was found in operation time (P = 0.190), intraoperative blood loss (P = 0.336), retrieved lymph nodes (P = 0.948), blood transfusion (P = 0.339), postoperative hospital stay (P = 0.194), or postoperative complications (P = 0.477) between the two groups. For overall survival, no statistically significant difference was found in all stages (P = 0.383), stage I (P = 0.431), stage II (P = 0.875) or stage III (P = 0.446) gastric cancer. Furthermore, regarding disease-free survival, no differences were found between the two groups in all stages (P = 0.378), stage I (P = 0.431), stage II (P = 0.879) or stage III (P = 0.510) gastric cancer.

**Conclusion:**

Age might not be an independent prognostic factor for short-term outcomes, OS, or DFS in gastric cancer patients who underwent gastrectomy. The pTNM stage of GC might be an independent prognostic factor for OS and DFS.

## Introduction

Gastric cancer (GC) is the fourth leading cause of cancer-related death in the world, and nearly 27,600 new cases were diagnosed in the USA in 2020 [1]. Despite persistent improvements in treatment strategies, GC is still considered an aggressive malignancy resulting in a poor prognosis, with a 5-year survival rate of 31% [2].

It is generally believed that the onset of GC mainly occurs in old patients [3]. However, the morbidity of GC in young patients has gradually increased over the past few years [4]. The definitions of young patients were different in previous studies, including the boundary ages of 30, 40 or 45 years old [5]. The unique challenges that are faced by young patients include psychosocial considerations of the family, the choice of fertility preservation, tolerance and adherence to cancer treatment, and unique genetic variations [6, 7].

Young patients were considered to have a poor prognosis compared with old patients in most studies. Lower differentiated histology and diffuse infiltration of the malignancy were found in young patients due to delayed detection [8, 9]. However, some studies reported that the overall survival (OS) of GC patients was not associated with age itself, but more prognostic factors that worked together [10, 11]. Unfortunately, there have been few studies providing the effect of age on GC patients using propensity score matching (PSM).

Thus, the purpose of the current study was to compare postoperative complications, OS and disease-free survival (DFS) in young and old gastric cancer patients after gastrectomy using PSM.

## Methods

### Patients

The medical records of 643 adult patients (aged ≥ 18 years) who underwent gastrectomy for gastric cancer in a single clinical database from January 2013 to December 2017 were enrolled continuously for retrospective analysis. The study was approved by the ethics committee of our institution (The First Affiliated Hospital of Chongqing Medical University, 2021-336), and all patients signed informed consent forms. This study was conducted in accordance with the World Medical Association Declaration of Helsinki as well.

### Inclusion and exclusion criteria

**Fig. 1:**
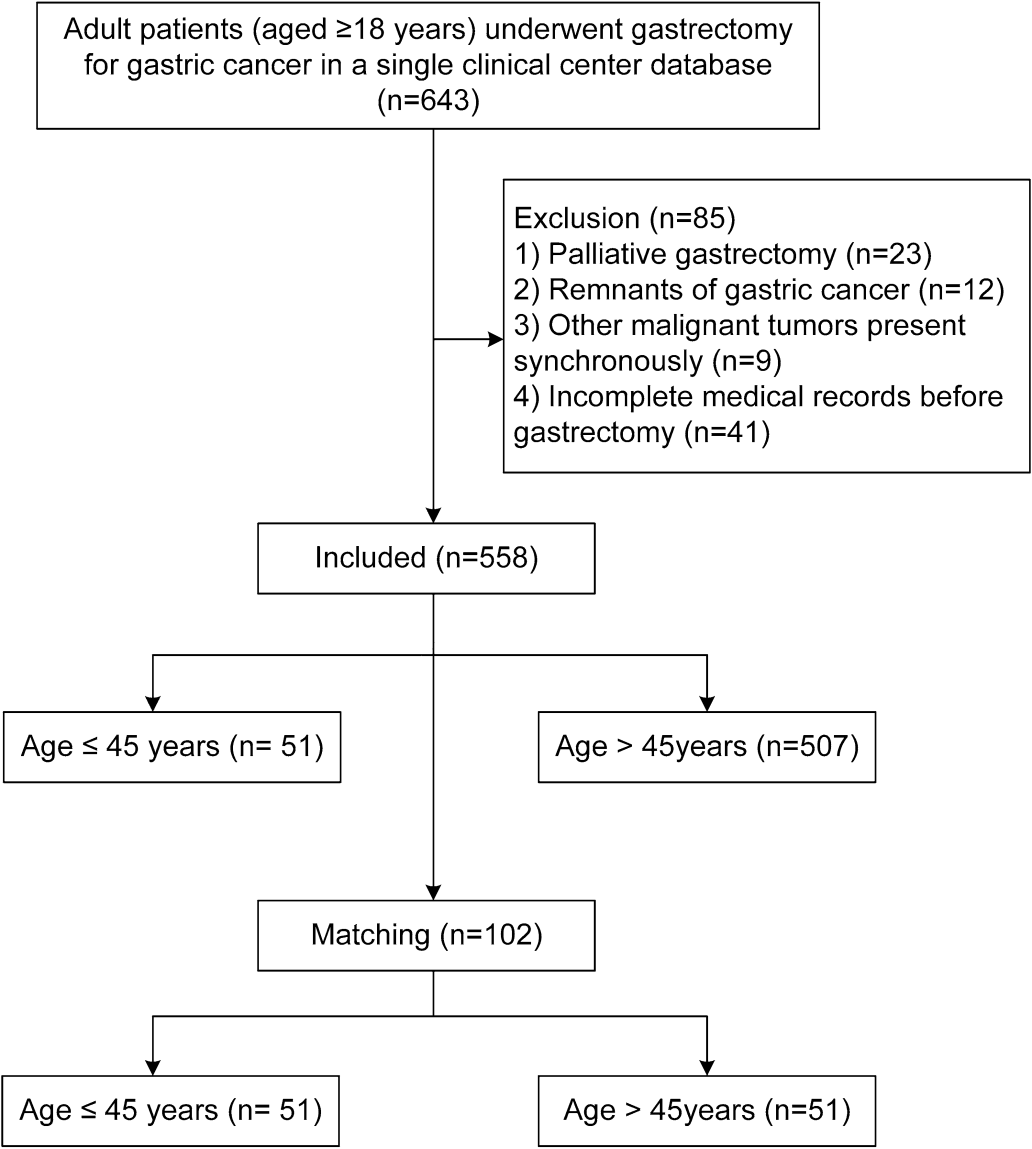
Flow chart of patient selection

Patients who were diagnosed pathologically with GC and undergoing gastrectomy were included in this study (n = 643). The exclusion criteria were as follows: (1) palliative gastrectomy (n = 23); (2) remnants of gastric cancer (n = 12); (3) other malignant tumors presented synchronously (n = 9); and (4) incomplete medical records before gastrectomy (n = 41). The flowchart and inclusion and exclusion criteria are shown in Fig. 1.

### Surgery management and follow-ups

According to the 5th edition guidelines of the Japanese Gastric Cancer Association (JGCA) in 2018, gastrectomy with D2 lymph node dissection is the standard treatment for patients with resectable GC [12]. The follow-ups of patients were strictly managed. Patients were followed up every 3 months with physical examinations and tumor markers for the first 2 years and every 6 months for the following 3 years. Patients were followed up every 6 months by computed tomography scans for the first 3 years and every year for the following 2 years, and they were followed up by endoscopy in the 1st year, 3rd year and 5th year [12].

### Definitions

The patients were divided into two groups: patients aged ≤ 45 years were considered the young group, and patients aged > 45 years were considered the old group. The pathological stages of gastric cancer in this study were defined by consulting by the 5th edition guidelines of the JGCA published in 2018 [12]. Postoperative complications were graded in accordance with the Clavien-Dindo classification [13]. According to the classification, major complications (grades ≥ III) required at least one of the following treatments: surgery, endoscopic intervention or radiological intervention [14]. OS was calculated by the time from gastrectomy to the death of the patient or the last follow-up. Disease-free survival (DFS) was defined as the time between gastrectomy and the first instance of tumor recurrence.

### Data collection

Perioperative information and follow-up data were collected for analysis. The perioperative information included baseline information, operation time, intraoperative blood loss, retrieved lymph nodes, postoperative hospital stay, type of resection, reconstruction methods, pTNM stage and postoperative complications. Follow-up data, including OS and DFS, were collected as well.

### Propensity score matching

To minimize the selection bias between the two groups, PSM was conducted in this study [15, 16]. Nearest neighbor matching was performed without replacement at a 1:1 ratio, and a caliper width with a 0.1 standard deviation (SD) was specified. The baseline information was matched, including sex, BMI, comorbidities, neoadjuvant chemotherapy, preoperative hemoglobin and albumin levels, type of resection, laparoscopy surgery, reconstruction methods, and pTNM stage.

### Statistical analysis

Continuous variables are expressed as the mean ± SD, and an independent-sample t test was used to compare the difference between the young and old groups. Frequency variables are expressed as n (%), and the Chi-square test or Fisher’s exact test was used. Cox regression analyses were performed to identify predictive factors for OS and DFS. Data were analyzed using SPSS (version 20.0) statistical software. A bilateral P value of < 0.05 was considered statistically significant.

## Results

### Baseline characteristics

A total of 643 adult patients (aged ≥ 18 years) who underwent gastrectomy were retrospectively analyzed in this study. According to the exclusion criteria, 558 patients were included in this study. There were 51 patients in the young group (aged ≤ 45 years) and 507 patients in the old group (aged > 45 years). Before PSM, there was a significant difference in the baseline characteristics, including sex (P = 0.000), comorbidities (P = 0.014), preoperative albumin levels (P = 0.000) and pTNM stage (P = 0.026), which are summarized in Table 1.

**Table 1:** Baseline characteristics before propensity score matching

Characteristics	Age ≤ 45 years (n = 51)	Age > 45 years (n = 507)	P value
Sex			0.000*
Male	22 (43.1%)	371 (73.2%)	
Female	29 (56.9%)	136 (26.8%)	
BMI (kg/m^2^)	21.9 ± 3.9	22.1 ± 3.1	0.751
Comorbidities	7 (13.7%)	152 (30.0%)	0.014*
Neo-adjuvant chemotherapy	4 (7.8%)	32 (6.3%)	0.560
Pre-operative hemoglobin (g/L)	124.5 ± 29.3	121.2 ± 26.9	0.418
Pre-operative albumin (g/L)	43.6 ± 4.9	39.7 ± 6.2	0.000*
Type of resection			0.632
Subtotal gastrectomy	34 (66.7%)	356 (70.2%)	
Total gastrectomy	17 (33.3%)	151 (29.8%)	
Laparoscopy surgery	49 (96.1%)	498 (98.2%)	0.266
Reconstruction methods			0.477
B I	21 (41.2%)	237 (53.8%)	
B II	9 (17.6%)	104 (20.5%)	
R-Y	21 (41.2%)	166 (32.7%)	
pTNM stage			0.026*
I	23 (45.1%)	147 (29.0%)	
II	13 (25.5%)	120 (23.7%)	
III	15 (29.4%)	240 (47.3%)	

BMI, body mass index; B-I, Billroth I reconstruction; B-II, Billroth II reconstruction; R-J, Roux-en-Y reconstruction; pTNM, pathological tumor node metastasis

Variables are expressed as the mean ± SD, n (%), *P-value < 0.05

### PSM analysis

After 1:1 matching according to PSM, 51 patients in the young group were matched to 51 patients in the old group. After PSM, there was no significant difference in any baseline characteristics between the two groups (Table 2).

**Table 2:** Baseline characteristics after propensity score matching

Characteristics	Age ≤ 45 years (n = 51)	Age > 45 years (n = 51)	P value
Sex			0.074
Male	22 (43.1%)	31 (60.8%)	
Female	29 (56.9%)	20 (39.2%)	
BMI (kg/m^2^)	21.9 ± 3.9	21.5 ± 3.4	0.584
Comorbidities	7 (13.7%)	9 (17.6%)	0.586
Neo-adjuvant chemotherapy	4 (7.8%)	7 (13.7%)	0.338
Pre-operative hemoglobin (g/L)	124.5 ± 29.3	121.7 ± 24.3	0.600
Pre-operative albumin (g/L)	43.6 ± 4.9	42.1 ± 7.9	0.274
Type of resection			0.413
Subtotal gastrectomy	34 (66.7%)	30 (60.8%)	
Total gastrectomy	17 (33.3%)	21 (39.2%)	
Laparoscopy surgery	49 (96.1%)	50 (98.0%)	1.000
Reconstruction methods			0.797
B-I	21 (41.2%)	18 (35.3%)	
B-II	9 (17.6%)	11 (21.6%)	
R-Y	21 (41.2%)	22 (43.1%)	
pTNM stage			0.318
I	23 (45.1%)	16 (31.4%)	
II	13 (25.5%)	14 (27.5%)	
III	15 (29.4%)	21 (41.2%)	

BMI, body mass index; B-I, Billroth I reconstruction; B-II, Billroth II reconstruction; R-J, Roux-en-Y reconstruction; pTNM, pathological tumor node metastasis

Variables are expressed as the mean ± SD, n (%), *P-value < 0.05

### Short-term outcomes

Short-term outcomes were compared between the two groups, and there were no differences found in operation time (P = 0.190), intraoperative blood loss (P = 0.336), retrieved lymph nodes (P = 0.948), blood transfusion (P = 0.339), postoperative hospital stay (P = 0.194), or postoperative complications. The postoperative complications were graded by Clavien-Dindo classification, and no differences were found in overall complications (P = 0.477) or major complications (P = 1.000) between the young group and the old group (Table 3).

**Table 3:** Short-term outcomes after propensity score matching

Characteristics	Age ≤ 45 years (n = 51)	Age > 45 years (n = 51)	P value
Operation time (minutes)	213.8 ± 53.7	227.0 ± 47.1	0.190
Intra-operative blood loss (mL)	137.5 ± 158.5	190.8 ± 360.0	0.336
Retrieved lymph nodes	22.2 ± 9.0	22.1 ± 9.2	0.948
Postoperative hospital stay (days)	11.6 ± 6.2	13.8 ± 10.4	0.194
Blood transfusion	4 (7.8%)	2 (3.9%)	0.339
Overall complications	13 (25.5%)	10 (19.6%)	0.477
Major complications	2 (3.9%)	3 (5.9%)	1.000

Variables are expressed as the mean ± SD, n (%)

### Overall survival

**Fig. 2:**
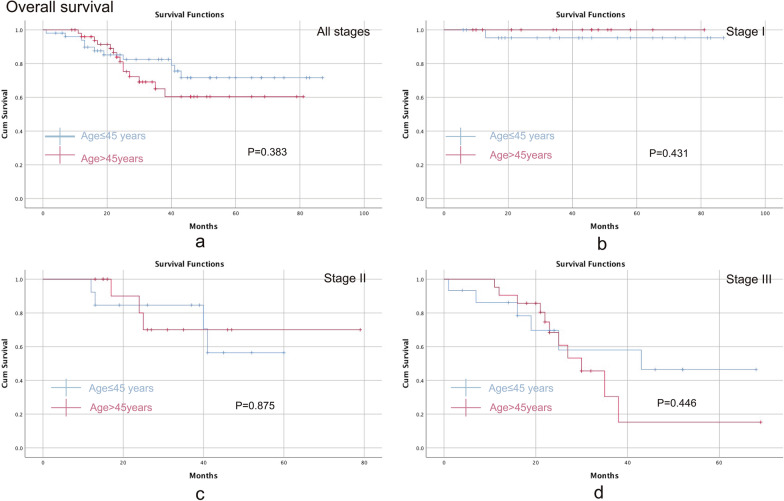
Comparison of the overall survival between the young group (aged ≤ 45 years) and the old group (aged > 45 years). **a** All stages; **b** stage I; **c** stage II; **d** stage III

The median follow-up time was 29.5 (1–87) months. The comparison of OS was performed between the young group and the old group. No statistically significant difference was found between the two groups in any stage (P = 0.383) (Fig. 2a). In the subgroup analysis of the stages, the young group showed no significant differences in stage I (P = 0.431) (Fig. 2b), stage II (P = 0.875) (Fig. 2c) or stage III (P = 0.446) (Fig. 2d) compared with the old group.

### Disease-free survival

**Fig. 3:**
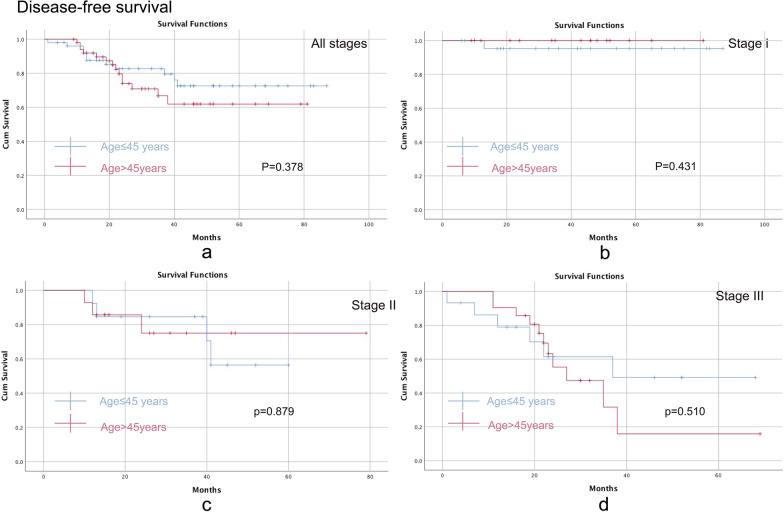
Comparison of disease-free survival between the young group (aged ≤ 45 years) and the old group (aged > 45 years). **a** All stages; **b** stage I; **c** stage II; **d** stage III

DFS was an important indicator to determine the potential role of age in recurrence. Similarly, there were no significant differences found between the two groups in any stage (Fig. 3a, P = 0.378) (Fig. 3a), stage I (P = 0.431) (Fig. 3b), stage II (P = 0.879) (Fig. 3c) or stage III (P = 0.510) (Fig. 3d).

### Cox regression analysis

Predictive risk factors were taken into consideration in multivariate Cox regression analysis. As a result, the pathological tumor node metastasis (pTNM) stage of malignancy was related to OS (P = 0.008) and DFS (P = 0.009), and age was not a predictor in terms of OS (P = 0.531) or DFS (P = 0.572) (Table 4).

**Table 4:** Multivariate analysis of OS and DFS

Risk factors	OS		DFS	
	HR (95% CI)	P value	HR (95% CI)	P value
Sex	0.905 (0.374–2.190)	0.825	0.900 (0.378–2.143)	0.812
Age	1.349 (0.529–3.439)	0.531	1.303 (0.520–3.269)	0.572
BMI	0.925 (0.780–1.908)	0.373	0.923 (0.779–1.903)	0.351
Comorbidities	1.246 (0.363–4.273)	0.726	1.094 (0.326–3.668)	0.884
Reconstruction methods	2.491 (0.949–6.537)	0.064	2.356 (0.924–6.005)	0.073
Type of resection	2.106 (0.500–8.871)	0.310	1.962 (0.479–8.040)	0.349
pTNM stage	2.587 (1.278–5.236)	0.008*	2.551 (1.265–5.145)	0.009*
Complications	1.096 (0.424–2.832)	0.850	1.245 (0.489–3.173)	0.646
Blood transfusion	2.997 (0.904–9.554)	0.063	2.893 (0.905–9.251)	0.073

OS, overall survival; DFS, disease-free survival; HR, hazard ratio; CI, confidence interval; BMI, body mass index; pTNM, pathological tumor node metastasis

^*^P-value < 0.05

## Discussion

A total of 558 patients, including 51 patients in the young group (aged ≤ 45 years) and 507 patients in the old group (aged > 45 years), were analyzed in this study. After 1:1 matching according to PSM, 51 patients in the young group were matched to 51 patients in the old group. After PSM, the baseline information of all the patients was not significantly different. In terms of postoperative complications, no difference was found in overall complications or major complications between the two groups. In addition, there were no significant differences in OS and DFS between the two groups.

Age was considered an important factor correlated with the prognosis of malignant tumors in previous studies [17]. Similarly, in GC patients, previous findings have demonstrated that the perioperative outcomes or OS were different in various age groups [8, 9]. GC patients were commonly classified into a young group and an old group; however, the cutoff value of age was different [8, 9]. In this study, we chose 45 years of age as the cutoff age for analysis, which was consistent with the majority of previous studies [18–20].

In addition, only a few studies have conducted PSM to control for selection bias [18], and it is still controversial whether age plays a role in OS and DFS after gastrectomy. PSM analysis is a statistical technique that can address confounding bias and mimic a randomized clinical trial, improving the level of evidence in studies [15, 21]. Thus, to avoid the confusing relationship between age and survival, this study carried out PSM to balance the baseline information.

Postoperative complications tend to directly affect the prognosis of patients who underwent gastrectomy, and major complications might also affect OS [22, 23]. It was believed in some previous studies that GC patients in the old group suffered more complications because of relatively poor cardiopulmonary conditions and uncontrolled comorbidities [24]. In this study, there was no difference in postoperative complications between the two groups, not only for overall complications but also for major complications. Thus, age was not considered a factor for complications after gastrectomy; however, postoperative comorbidities, including hypertension and diabetes, tumor site, aortic calcification, and pathological staging of the tumor, contributed more to the complications [22, 25].

It is widely accepted that the OS of GC patients after gastrectomy is the major outcome indicator to determine the effectiveness of treatment [26]. There is still controversy about the effectiveness of age on OS. Some studies have reported a lower OS in old GC patients because of more complications [24]; however, other studies showed that young patients have a much shorter survival time than old patients [9, 27]. Large tumors and poorly differentiated tumors accounted for a high incidence of peritoneal recurrence [27–29]. Moreover, Seo et al. reported that early onset GC had more aggressive features [30]. Koea et al. described genetic susceptibility in young patients, with familial clustering up to 19% [31]. However, OS in the young group was not significantly different from that in the old group in this study, which was consistent with a previous study [32]. This is probably because all the baseline information was matched through the PSM analysis, and the selection bias declined maximally. Another reason might be the relatively small sample size.

The quality of life of patients with malignant tumors depends mostly on DFS, and the recurrence of tumors can result in difficult treatments and imminent death. Young patients might experience more cancer-related deaths, and the causes of death in old patients might be closely related to other comorbidities [9]. Notably, a lower tolerance for radical gastrectomy in old patients contributed more to lower 5-year survival than tumor recurrence [33, 34]. Interestingly, this is not similar to our study, and the findings revealed that the young group had a comparable survival time before recurrence compared to the old group. Moreover, the pTNM stage of GC was found to be the only independent prognostic factor in GC patients in terms of OS and DFS.

This current study had several strengths. First, this study was carried out using PSM to minimize the selection bias caused by baseline information, and subgroup analysis was conducted for each tumor stage to analyze OS and DFS. Second, previous PSM studies reported that age had a significant impact on survival; however, in this study, no significant difference was found.

There were some limitations in the current study. First, this is a single-center retrospective study with a small sample size, which might cause bias. Second, to decrease the selection bias on baseline information, PSM analysis was conducted in this study; however, another bias existed due to the smaller sample size in the subgroup analysis of different stages. Third, the median follow-up time was relatively short, especially for the early stage of tumors. Thus, a larger sample size and multicenter prospective randomized controlled trials should be conducted in the future.

In conclusion, age might not be an independent prognostic factor for short-term outcomes, OS, or DFS in gastric cancer patients who underwent gastrectomy. The pTNM stage of GC might be an independent prognostic factor for OS and DFS.

## Data Availability

The datasets used and analyzed during the current study are available from the corresponding author on reasonable request.
